# Morphogenesis and morphometric scaling of lung airway development follows phylogeny in chicken, quail, and duck embryos

**DOI:** 10.1186/s13227-016-0049-3

**Published:** 2016-05-26

**Authors:** Daniel Tzou, James W. Spurlin, Amira L. Pavlovich, Carolyn R. Stewart, Jason P. Gleghorn, Celeste M. Nelson

**Affiliations:** Department of Chemical and Biological Engineering, Princeton University, 303 Hoyt Laboratory, William Street, Princeton, NJ 08544 USA; Department of Molecular Biology, Princeton University, 303 Hoyt Laboratory, William Street, Princeton, NJ 08544 USA

**Keywords:** Morphodynamics, Patterning, Lateral branching, Allometry

## Abstract

**Background:**

New branches within the embryonic chicken lung form via apical constriction, in which epithelial cells in the primary bronchus become trapezoidal in shape. These branches form at precise locations along the primary bronchus that scale relative to the size of the organ. Here, we examined the extent to which this scaling relationship and branching mechanism are conserved within lungs of three species of birds.

**Findings:**

Analyzing the development of embryonic lungs from chicken, quail, and duck, as well as lungs explanted and cultured ex vivo, revealed that the patterns of branching are remarkably conserved. In particular, secondary bronchi form at identical positions in chicken and quail, the patterns of which are indistinguishable, consistent with the close evolutionary relationship of these two species. In contrast, secondary bronchi form at slightly different positions in duck, the lungs of which are significantly larger than those of chicken and quail at the same stage of development. Confocal analysis of fixed specimens revealed that each secondary bronchus forms by apical constriction of the dorsal epithelium of the primary bronchus, a morphogenetic mechanism distinct from that used to create branches in mammalian lungs.

**Conclusions:**

Our findings suggest that monopodial branching off the primary bronchus is driven by apical constriction in lungs of chicken, quail, and duck. The relative positions at which these branches form are also conserved relative to the evolutionary relationship of these species. It will be interesting to determine whether these mechanisms hold in more distant species of birds, and why they differ so significantly in mammals.

## Findings

### Background

 The vertebrate lung develops from a ventral outpouching of foregut endoderm, a process that begins at embryonic day (*E*) 4 in the chicken, *E*9 in the mouse, and *E*26 in the human [1, 2]. This anlage forms the lung buds, which in mammals develop into the primary bronchi that subsequently undergo recursive rounds of lateral and dichotomous branching to form the airway epithelial tree of the bronchoalveolar lung [3, 4]. In birds, the primary bronchi develop secondary bronchi via monopodial branching to generate the airways of the parabronchial lung [5]. The tertiary bronchi (parabronchi), which conduct air continuously in one direction in the avian lung, later anastomose and establish the air capillaries [6]. Morphogenesis of the airways has been examined extensively in mice, where it is thought to be driven by fibroblast growth factor (FGF)-10-mediated induction of epithelial proliferation and chemotaxis [4]; this mechanism is considered to be conserved across vertebrates [7]. Nonetheless, we recently found that in the chicken, monopodial branching is driven by apical constriction of the airway epithelium [8]. Amazingly, each new secondary bronchus forms at a precise location along the length of the chicken primary bronchus, a location that scales relative to the size of the lungs [5]. Whether this morphogenetic mechanism and morphometric scaling are conserved in other avian species is unknown.

### Results

#### Patterns of monopodial branching in lungs from chicken, quail, and duck

**Fig. 1:**
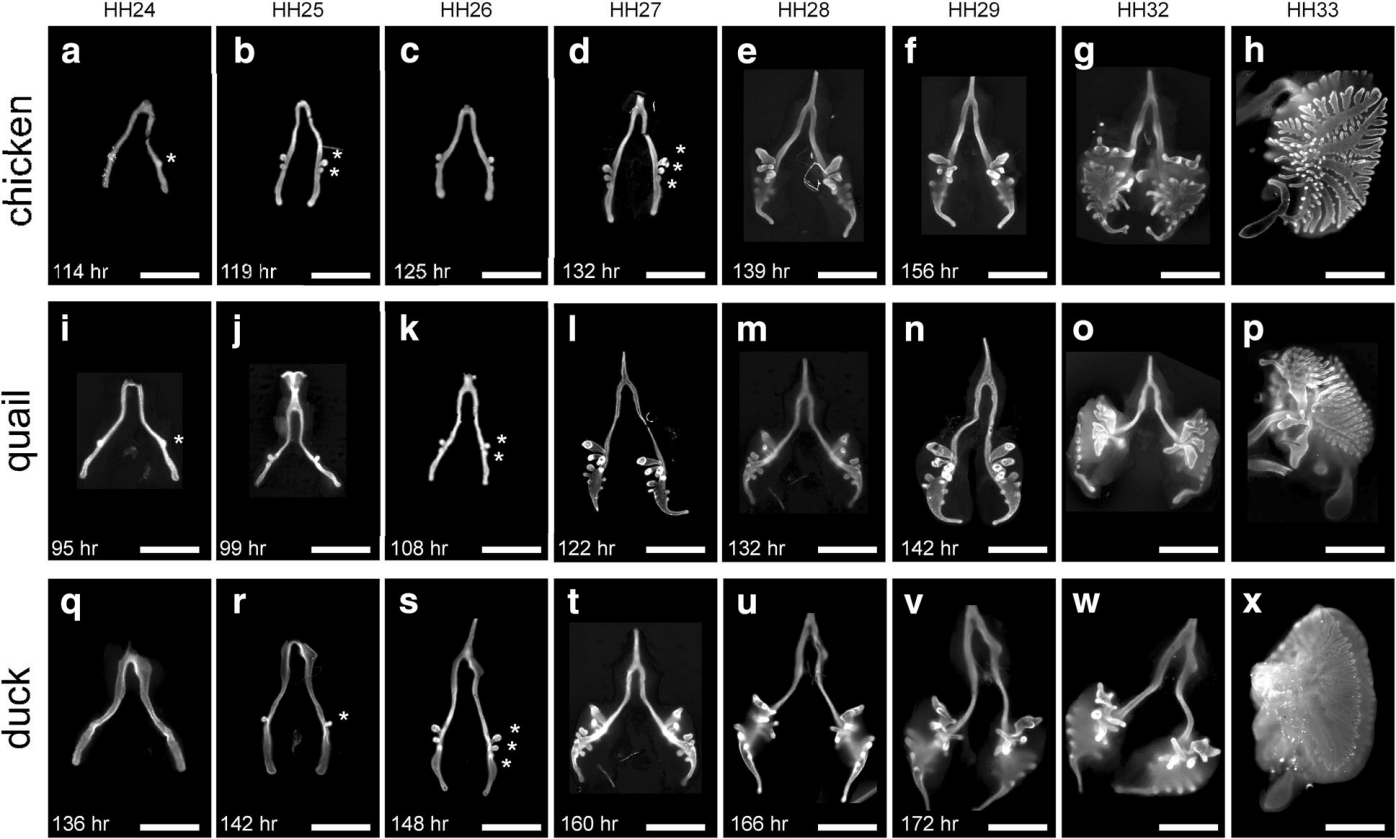
Monopodial branching of the embryonic lungs of (**a**–**h**) domestic chicken *Gallus gallus*, (**i**–**p**) Japanese quail *Coturnix japonica*, or (**q**–**x**) pekin duck *Anas platyrhynchos*. Shown are fluorescence images of E-cadherin staining at different HH stages, with time of incubation indicated in *lower left corner* of figure *panel*. *Scale bars* 500 μm. Branches are denoted by (*asterisk*)

**Fig. 2:**
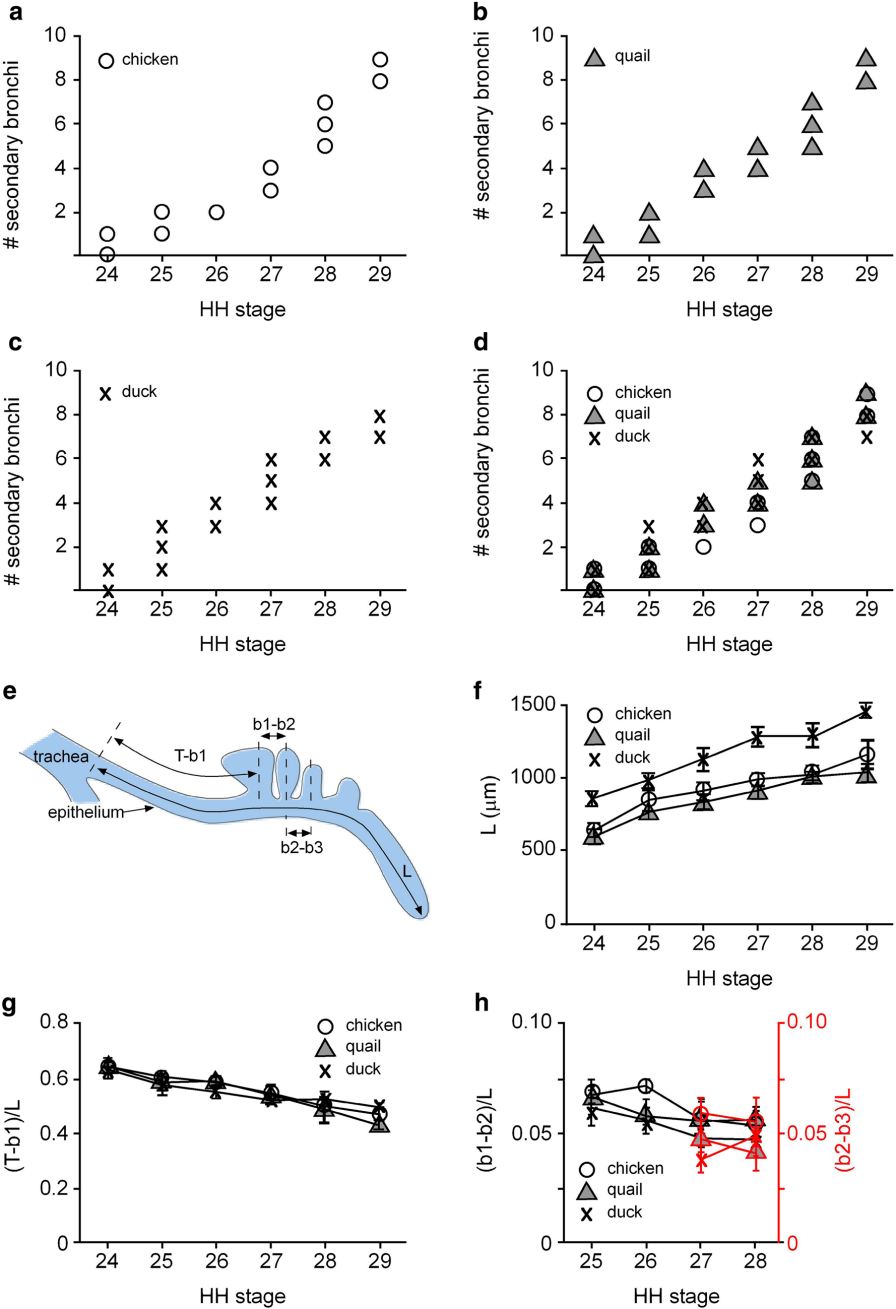
Morphometric analysis of lungs as a function of developmental time (Hamburger–Hamilton stage) in chicken, quail, and duck embryos. Shown are (**a**–**d**) number of secondary bronchi in each species. **e** Schematic of the relative positions of the secondary bronchi (*b1*, *b2*, and *b3*) as a function of the length of the primary bronchus (*L*), as measured from the point of the tracheal bifurcation (*T*). Using this framework, we measured (**f**) *L* and (**g**, **h**) the relative positions of secondary bronchi in chicken, quail, and duck lungs. Shown are mean ± SD

In the embryonic chicken lung, the first secondary bronchus (*b1*) forms on the dorsal surface of the primary bronchus at Hamburger–Hamilton stage (HH) 24 (Fig. 1a). The second (*b2*) appears just distal to *b1* at HH25 (Fig. 1b) and the third (*b3*) emerges distal to *b2* at HH27, in a slightly more ventral position along the primary bronchus than the first two branches (Fig. 1c, d). By HH28, *b1*–*b3* extend into the surrounding mesenchyme, and three to four additional secondary bronchi have branched off each primary bronchus (Figs. 1e, 2a, d). Subsequently, parabronchi begin to form and extend toward each other by HH33 (Fig. 1f–h).

Japanese quail and domestic chickens diverged approximately 35 m.y.a. [9, 10]; both are in the same order (Galliformes) and family (Phasianidae) of birds. Ducks belong to the order Anseriformes and family Anatidae and are estimated to have diverged from Galliformes approximately 90 m.y.a. [11]. We examined lungs from staged quail [12] and Pekin duck [13, 14] embryos and found that *b1* also forms on the dorsal surface of the primary bronchus at HH24 (Fig. 1i–x). *b2* appears just distal to *b1* by HH26. By HH29, an average of eight secondary bronchi are apparent on the primary bronchi of both species (Fig. 2b–d). The secondary bronchi then generate parabronchi that also start extending by HH33.

#### Quantitative morphometric analysis of branch positions in avian lungs

In our previous work examining the signaling that controls monopodial branching of the embryonic chicken lung, we found that secondary bronchi formed at precise positions along the primary bronchus in cultured lung explants [5]. To examine whether these positions are conserved across the three species *in ovo*, we used morphometric analysis to quantify the length of each primary bronchus (*L*) and the relative positions of *b1*, *b2*, and *b3* (Fig. 2e) as a function of HH stage. We found that *L* is essentially the same for chicken and quail at HH24, but ~30 % longer in duck; *L* increases at approximately the same rate for chicken (~8 μm/hr) and quail (~7 μm/hr) and faster in the duck (~16 μm/hr) (Fig. 2f). The position of *b1* scales with lung size, first emerging ~65 % down the length of the primary bronchus at HH24, as measured from the tracheal bifurcation (*T*–*b1* = *0.65L*) (Fig. 2g). This relative distance decreases over developmental time, which suggests that most of the growth of the primary bronchus occurs at its distal end. Consistently, *b2* forms in a stereotyped location that is identical in chicken and quail: The distance between *b1* and *b2* is ~7 % the length of the primary bronchus (*b1*–*b2* = *0.07L*_chicken_ = *0.07L*_quail_). In contrast, *b2* forms slightly closer to *b1* in the duck (*b1*–*b2* = *0.06L*_duck_), and this difference persists over time (Fig. 2h). These distances decrease modestly through HH28. Similarly, the formation of *b3* is stereotyped at ~5 % the length of the primary bronchus, a distance that does not change appreciably from HH27 to HH28.

#### Morphometric analysis of lung explants from chicken, quail, and duck

**Fig. 3:**
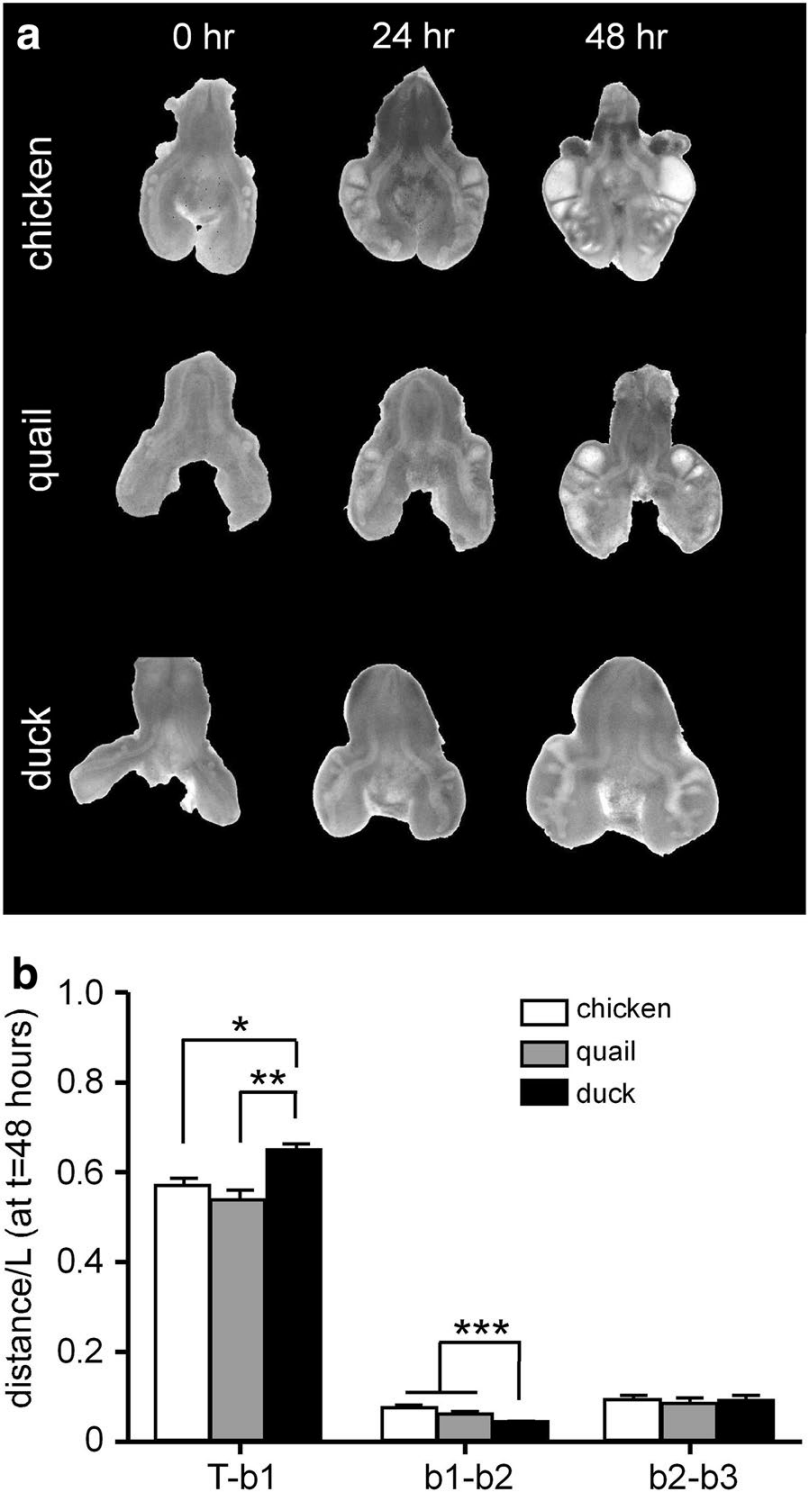
**a** Secondary bronchi continue to form in lung explants cultured ex vivo. Shown are bright-field images for lungs of each species at 0, 24, and 48 h of culture. **b** Quantification of the relative positions of secondary bronchi in lung explants from chicken, quail, and duck. Shown are mean ± SEM for *n*_chicken_ = 9, *n*_quail_ = 8, and *n*_duck_ = 5. *p < 0.05; **p < 0.01, ***p < 0.001, as determined by one-way ANOVA with Tukey’s posttest

We performed a similar analysis on cultured lungs explanted from HH24-25-stage chicken, quail, or duck embryos, which initially had one or two secondary bronchi on each primary bronchus (Fig. 3a). After 48 h of culture, *b1* emerges at an identical position in chicken and quail explants (*T*–*b1* = *0.57L*_chicken_ = *0.54L*_quail_), which are approximately the same size (*L*_quail_ = 0.97*L*_chicken_). In contrast, *b1* forms at a position significantly more distal in the duck explants (Fig. 3b), ~65 % down the length of the primary bronchus (*T*–*b1* = *0.65L*_duck_), which is also significantly longer (*p* < 0.03) at this stage (*L*_duck_ = *1.14L*_chicken_).

Similar to the data *in ovo*, the branching patterns of the chicken and quail lung explants are indistinguishable. In both, the distance between *b1* and *b2* is ~6 % the length of the primary bronchus (*b1*–*b2* = *0.07L*_chicken_, *0.06L*_quail_). Similarly, the distance between *b2* and *b3* is ~9 % the length of the primary bronchus (*b2*–*b3* = *0.09L*_chicken_, *0.09L*_quail_). In contrast, the position of *b2* diverged slightly, but significantly (*p* < 0.001), in the duck, with *b1*–*b2* = *0.04L*_duck_ and *b2*–*b3* = *0.09L*_duck_. These data suggest that the relative positions of *b1*, *b2*, and *b3* are conserved across chicken and quail, and very similar in duck, consistent with the evolutionary relationships between these three species of birds.

**Fig. 4:**
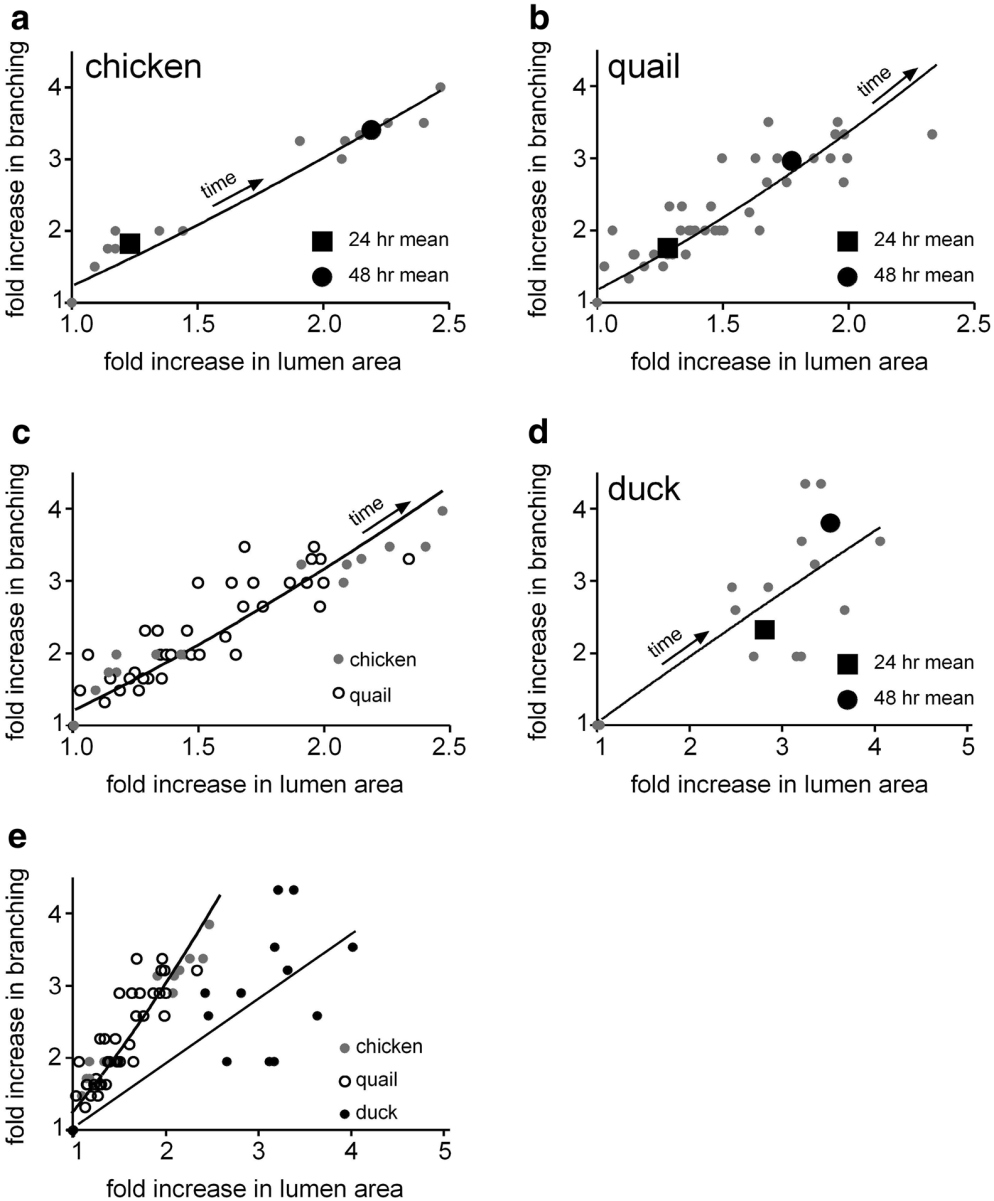
Allometric analysis of development of lung explants. Shown are data from individual explants at different time points, as well as the 24- and 48-h mean for **a** chicken, **b** quail, **c** chicken and quail, **d** duck, and **e** all three species. *Curves* depict power-law fits for each set of data

To compare rates of branching quantitatively, we compared the fold-change in extent of branching to fold-change in the projected area of the lumen of the developing airways [5]. For the chicken lung explants, we observed an approximate doubling in branching by 24 h of culture with ~30 % increase in luminal area (Fig. 4a). By 48 h of culture, the explants had a more than threefold increase in branching compared to time zero, with a doubling in luminal area. These data collapsed onto a single curve that could be fitted to a power-law model ($y \propto x^{1.29}$; *R*^*2*^ = 0.95), suggesting that development is stereotyped in embryonic chicken lung explants.

We observed more spread in the data from quail lung explants (Fig. 4b). Nonetheless, development in the quail could also be described by a power-law model ($y \propto x^{1.51}$; *R*^2^ = 0.87). Explanted quail lungs developed more slowly than those of chicken, with slightly reduced rates of both branching and luminal growth. Even so, development of both chicken and quail lungs could be described by the same curve ($y \propto x^{1.39}$; *R*^2^ = 0.88) (Fig. 4c), consistent with the lungs of these species following the same developmental trajectory, albeit at different rates. In contrast, the morphogenesis of duck lung explants was significantly different (Fig. 4d), with a poor fit to a distinct power-law model ($y \propto x^{0.93}$; *R*^*2*^ = 0.7). These data may suggest that the rate of development of the duck lung in culture is less stereotyped than that of the other two species (Fig. 4e).

#### Apical constriction is conserved during monopodial branching of avian lungs

**Fig. 5:**
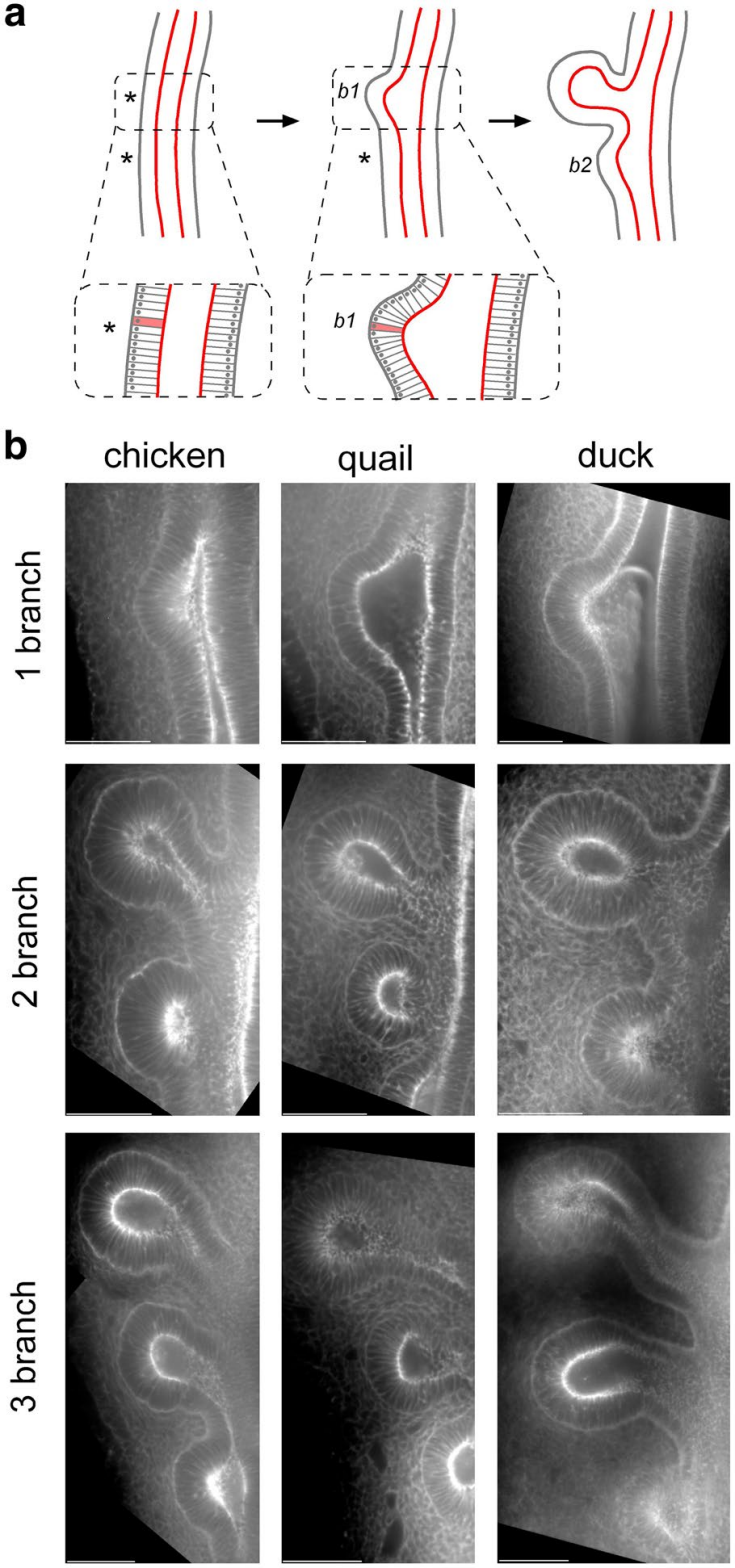
**a** Apical constriction of the primary bronchus drives formation of the secondary bronchi. The apical surface (*red line*) of cells in the branching region contracts, transforming the cell from rectangular to trapezoidal in cross section (*insets*). **b** Confocal sections of phalloidin-stained lungs of chicken, quail, and duck embryos at the one-branch (*top*), two-branch (*middle*), and three-branch (*bottom*) stages. *Scale bars* 50 μm

In contrast to the prevailing model in the field that airway branching is driven by differential proliferation [15], we recently found that secondary bronchi branched off the surface of the primary bronchus of the embryonic chicken via cell shape changes mediated by apical constriction of the epithelium (Fig. 5a) [8]. Embryonic lungs were stained for F-actin and imaged by confocal microscopy. Three-dimensional reconstruction of the confocal stacks revealed that the epithelium of the primary bronchus of the embryonic chicken lung is essentially columnar in geometry (Fig. 5b). Additionally, F-actin is concentrated at the apical surface of the airway epithelium as *b1* emerges from the dorsal surface of the primary bronchus, consistent with apical constriction of the epithelium. We found a similar morphology of the airway epithelium and pattern of F-actin localization in lungs from quail and duck embryos (Fig. 5b), with apical constriction evident during the formation of all three branches in both species.

### Conclusions

To the best of our knowledge, this report provides the first quantitative morphometric analysis of lung development across avian species. Our results suggest that apical constriction might be conserved in birds, where it appears to be induced by FGF10 [8], which has been reported to be expressed focally in the subjacent mesenchyme in the embryonic chicken lung [7]. In mammals, a similar pattern of FGF10 expression [16, 17] was proposed to induce branching by elevating proliferation of the epithelium [18]; our data thus imply that signaling downstream of FGF10 is distinct in birds and mice [19]. Our results also suggest that the locations of branches scale across lungs, both within a species as we found previously [5], as well as between species. Importantly, more closely related species show more similar morphometry of the growing airways, suggesting that evolutionary connections between birds can be observed even at the level of lung organogenesis.

### Methods

#### Incubation and immunofluorescence analysis

Fertilized chicken (*Gallus gallus* variant *domesticus*, White Leghorn), pekin duck (*Anas platyrhynchos domestica*), and Japanese quail (*Coturnix japonica*) eggs were obtained from Hyline International, www.duckeggs.com or Metzer Farms, and G.Q.F. Manufacturing Company Inc, respectively, and handled following Princeton IACUC-approved protocol #1934. Upon receipt, embryonated eggs were incubated at 38 °C in a humidified chamber. Whole embryonic lungs were dissected, fixed, and stained for E-cadherin and F-actin, as described [8].

#### Ex vivo culture of embryonic lungs

Whole embryonic lungs were dissected at HH24-25 in PBS supplemented with 100 U/mL penicillin–streptomycin (Invitrogen). After dissection, two or three explants were placed on a membrane (11-μm-diameter pore; Whatman) floating on BGJb medium (Invitrogen) supplemented with 100 U/mL penicillin–streptomycin (Invitrogen) and 0.2 mg/mL ascorbic acid (Sigma) [20]. Lungs were cultured for 48 h under optimal humidity at 37 °C in 5 % CO_2_, and medium was changed every 24 h.

#### Morphometric analysis

Bright-field images were acquired with a stereomicroscope (Olympus), and secondary bronchi were enumerated. As a metric of the relative size of the airways, images were thresholded and a two-dimensional projection of the area of the lumen was measured using ImageJ. Fold-change in number of branches and projected luminal area (compared to time zero) were used to describe the relationship between branching and growth as a function of time, as described [5].

## Abbreviations

*E*: embryonic day
FGF: fibroblast growth factor
HH: Hamburger–Hamilton

## References

[1] Warburton D, Bellusci S, De Langhe S, Del Moral PM, Fleury V, Mailleux A, Tefft D, Unbekandt M, Wang K, Shi W (2005). Molecular mechanisms of early lung specification and branching morphogenesis. Pediatr Res.

[2] Locy WA, Larsell O (1916). The embryology of the bird’s lung based on observations of the domestic fowl, part I. Am J Anat.

[3] Metzger RJ, Klein OD, Martin GR, Krasnow MA (2008). The branching programme of mouse lung development. Nature.

[4] Morrisey EE, Hogan BL (2010). Preparing for the first breath: genetic and cellular mechanisms in lung development. Dev Cell.

[5] Gleghorn JP, Kwak J, Pavlovich AL, Nelson CM (2012). Inhibitory morphogens and monopodial branching of the embryonic chicken lung. Dev Dyn.

[6] Maina JN (2005). The lung-air sac system of birds.

[7] Moura RS, Coutinho-Borges JP, Pacheco AP, Damota PO, Correia-Pinto J (2011). FGF signaling pathway in the developing chick lung: expression and inhibition studies. PLoS ONE.

[8] Kim HY, Varner VD, Nelson CM (2013). Apical constriction initiates new bud formation during monopodial branching of the embryonic chicken lung. Development.

[9] van Tuinen M, Dyke GJ (2004). Calibration of galliform molecular clocks using multiple fossils and genetic partitions. Mol Phylogenet Evol.

[10] van Tuinen M, Hedges SB (2001). Calibration of avian molecular clocks. Mol Biol Evol.

[11] Hackett SJ, Kimball RT, Reddy S, Bowie RC, Braun EL, Braun MJ, Chojnowski JL, Cox WA, Han KL, Harshman J (2008). A phylogenomic study of birds reveals their evolutionary history. Science.

[12] Ainsworth SJ, Stanley RL, Evans DJ (2010). Developmental stages of the Japanese quail. J Anat.

[13] Dupuy V, Nersessian B, Bakst MR (2002). Embryonic development from first cleavage through seventy-two hours incubation in two strains of pekin duck (*Anas platyrhynchos*). Poult Sci.

[14] Sellier N, Brillard J-P, Dupuy V, Bakst MR (2006). Comparative staging of embryo development in chicken, turkey, duck, goose, guinea fowl, and Japanese quail assessed from five hours after fertilization through seventy-two hours of incubation. J Appl Poultry Res.

[15] Miura T (2015). Models of lung branching morphogenesis. J Biochem.

[16] Bellusci S, Grindley J, Emoto H, Itoh N, Hogan BL (1997). Fibroblast growth factor 10 (FGF10) and branching morphogenesis in the embryonic mouse lung. Development.

[17] Park WY, Miranda B, Lebeche D, Hashimoto G, Cardoso WV (1998). FGF-10 is a chemotactic factor for distal epithelial buds during lung development. Dev Biol.

[18] Weaver M, Dunn NR, Hogan BL (2000). Bmp4 and Fgf10 play opposing roles during lung bud morphogenesis. Development.

[19] Varner VD, Nelson CM (2014). Cellular and physical mechanisms of branching morphogenesis. Development.

[20] Trowell OA (1959). The culture of mature organs in a synthetic medium. Exp Cell Res.

